# A new species of *Queskallion* Smetana, 2015 (Coleoptera, Staphylinidae, Quediina) from China

**DOI:** 10.3897/zookeys.945.53480

**Published:** 2020-07-03

**Authors:** Yanpeng Cai, Xiaoyan Li, Hongzhang Zhou

**Affiliations:** 1 Morphological Laboratory, Guizhou University of Traditional Chinese Medicine, Guiyang, 550025, Guizhou, China Guizhou University of Traditional Chinese Medicine Guiyang China; 2 Key Laboratory of Zoological Systematics and Evolution, Institute of Zoology, Chinese Academy of Sciences, 1 Beichen West Rd., Chaoyang District, Beijing 100101, China Langfang Normal University Langfang China; 3 University of the Chinese Academy of Sciences, 19A Yuquan Rd., Shijingshan District, Beijing, 100049, China Institute of Zoology Beijing China; 4 Hebei Key Laboratory of Animal Diversity, Langfang Normal University Aiminxidao 100, Anci Area, Langfang 065000, Hebei Province, China University of the Chinese Academy of Sciences Beijing China

**Keywords:** Checklist, key, morphology, rove beetle, taxonomy

## Abstract

A new species, *Queskallion saetosum***sp. nov.**, is described herein from Sichuan Province, China. It is diagnosed from a closely related species, *Q. tangi* Smetana, 2015. Color images and line drawings of the adult of the new species, as well as its genitalia are provided. In addition, a checklist of species, an updated key to species and a geographical distribution map of all known species in the genus *Queskallion* Smetana are included.

## Introduction

The genus *Queskallion* was established by Smetana (2015) in the subtribe Quediina sensu stricto (Brunke et al. 2016), with *Queskallion tangi* Smetana, 2015 as the type species by original designation. It is a small genus including only five species, known from China, Myanmar and Nepal.

The genus *Queskallion* is mainly characterized by the second and third segments of the antenna. In addition to the usual long setae, they have numerous short setae and the surface between the setae is slightly granulose, not quite shiny, therefore visually not obviously contrasting with the dull granulose surface of the following segments bearing dense appressed pubescence; the pronotum with three to five additional setiferous punctures on the posterior lateral area well behind the large lateral puncture; and the surface of the elytra with characteristic semigranulose microsculpture giving it a greasy appearance (Smetana 2015).

Examination of rove beetle specimens collected from Sichuan Province uncovered the new species. This study aims to describe the new species, distinguish it from congeners and provide up-to-date information on the distribution of the genus (Fig. 3).

## Material and methods

Specimens were relaxed in warm water (60 °C) for 5–8 hours for dissection of the abdominal segments VIII–X and the genitalia. After examination, the dissected body parts were glued back to the mounting cards for future study. Observation, dissection and measurements were performed using a stereo microscope (Zeiss SteREO Discovery V20). Images of the adult and genitalia were captured with an AxioCam MRc 5 camera attached to a Zeiss Axio Zoom V16 Fluorescence Stereo Zoom Microscope, and photomontage was performed in Zen 2012 (blue edition) imaging software (https://www.zeiss.com.cn/microscopy/products/microscope-software/zen.html). Inkscape V0.91 was used to make the line drawings. The abdominal tergites and sternites were entirely flattened for the line drawings to make the illustrations more distinguishable among species. Species distribution data were compiled within Microsoft Excel using both published records and specimen label data. The distribution map was produced with the aid of DIVA-GIS 7.5 (Hijmans et al. 2011).

The specimens examined, including types, were deposited in the Institute of Zoology, Chinese Academy of Sciences (IZ-CAS).

Morphological terminology followed Smetana and Davies (2000) and Smetana (2015).

The following abbreviations are used in the text:

**BL** body length (from apex of clypeus to apex of abdominal tergite VIII);

**BW** body width (maximal body width, usually equal to EW);

**HL** head length (from base of clypeus to neck constriction);

**HW** head width (maximal head width, including eyes);

**PL** pronotal length (along midline of pronotum);

**PW** pronotal width (maximal pronotal width);

**EL** elytral length (maximal elytral length);

**EW** elytral width (maximal elytral width);

**ESL** elytral suture length (from apex of scutellum to apex of elytral suture);

**AW** abdominal width (maximal width of abdomen);

**HEL** (head) eye length;

**HTL** (head) temporal length.

## Taxonomy

### Queskallion

Taxon classificationAnimaliaColeopteraStaphylinidae

Smetana, 2015: 399.

731EF68A-FCFB-5D28-A880-6C387E720826

#### Type species.

*Queskallion tangi* Smetana, 2015, by original designation.

##### Checklist of *Queskallion* Smetana species

*Queskallion dispersepunctatum* (Scheerpeltz, 1965: 209)

Distribution: China (Yunnan Province), Myanmar, Nepal.

*Queskallion montanum* Smetana, 2015: 410

Distribution: China (Gansu and Sichuan Provinces).

*Queskallion saetosum* sp. nov.

Distribution: China (Sichuan Province).

*Queskallion schuelkei* Smetana, 2015: 408

Distribution: China (Yunnan Province).

*Queskallion seronatum* Smetana, 2015: 411

Distribution: Nepal.

*Queskallion tangi* Smetana, 2015: 407

Distribution: China (Xizang Autonomous Region).

### Key to the species of *Queskallion* Smetana

(updated from Smetana (2015) to include the new species)

**Table:** 

1	Last puncture of dorsal rows on pronotum shifted considerably posteriad toward posterior third of pronotal length, each dorsal row with four punctures. Paramere of aedeagus quite narrow and elongate; sensory peg setae on underside not numerous, most situated near apex of paramere	***Q. dispersepunctatum* (Scheerpeltz)**
–	Last puncture of dorsal rows on pronotum not shifted posteriad, situated before or at middle of pronotal length, each dorsal row with three punctures. Paramere of aedeagus of different shape, in general much wider, sensory peg setae on underside numerous and located differently	**2**
2	Sensory peg setae on underside of paramere arranged into groups, forming certain characteristic figures	**3**
–	Sensory peg setae on underside of paramere widely spread over most of the fusiform part of paramere, not forming any kind of discernible figure	**4**
3	Paramere of aedeagus slightly narrower than median lobe (Fig. 2G), underside with sensory peg setae arranged into one characteristic inversed Y-shaped figure (Figs 1D, 2E). Female tergite X with apical margin forming distinctive M-shaped indention (Fig. 2H)	***Q. saetosum* sp. nov.**
–	Paramere of aedeagus with subapical portion slightly dilated laterally, becoming wider than median lobe, underside with sensory peg setae arranged into two S-like figures. Female tergite X with apical margin deeply and arcuately emarginated	***Q. tangi* Smetana**
4	Apical portion of median lobe of aedeagus parallel-sided with apex slightly emarginated, aedeagus in general relatively short	***Q. schuelkei* Smetana**
–	Apical portion of median lobe of aedeagus at least slightly narrowed toward arcuate apex, aedeagus in general longer	**5**
5	Aedeagus in general quite narrow, elongate, apical portion of paramere moderately dilated, elongate-oval in shape	***Q. seronatum* Smetana**
–	Aedeagus in general broader, less elongate, apical portion of paramere markedly dilated, broadly-oval in shape	***Q. montanum* Smetana**

### Queskallion saetosum sp. nov.

Taxon classificationAnimaliaColeopteraStaphylinidae

F3DC5D77-8F47-5FD1-8DA4-073D86D95CB1

http://zoobank.org/DA7B0E5D-4B45-4BC4-AF10-1CCE5D81AA02

Figures 1 , 2

#### Material examined.

***Holotype***: China • ♂; Sichuan Province, Baoxing County, Longdong, Ruobigou; alt. 1600 m; 10 August 2003; Xiaodong Yu (IZ-CAS) leg. ***Paratypes***: 3 ♂♂, 1 ♀; same locality as holotype; 10−13 August 2003; Xiaodong Yu (IZ-CAS) leg.

#### Diagnosis.

This new species is very similar to *Q. tangi* Smetana in all characters, but it can be distinguished from the latter by having the male paramere of the aedeagus slightly narrower than the median lobe, underside with sensory peg setae arranged into one characteristic inversed Y-shaped figure, female tergite X with apical margin forming distinctive M-shaped indention; whereas the latter has male paramere of aedeagus with subapical portion slightly dilated laterally becoming wider than median lobe, underside with sensory peg setae arranged into two S-like figures, female tergite X with apical margin deeply and arcuately emarginated.

#### Description.

Head dark brown to blackish brown; pronotum, scutellum and elytra dark brown; abdomen dark brown, each tergite with posterior margin slightly paler; head, pronotum and abdomen strongly iridescent; antennae dark brown, labrum yellowish-brown, mandibles dark brown, maxillary and labial palpi dark brown; legs dark brown.

BL = 8.5 mm, BW = 1.7 mm, HL/PL/EL = 1.00: 1.52: 1.80, HW/PW/EW/AW = 1.00: 1.60: 1.84: 1.55.

**Figure 1.**
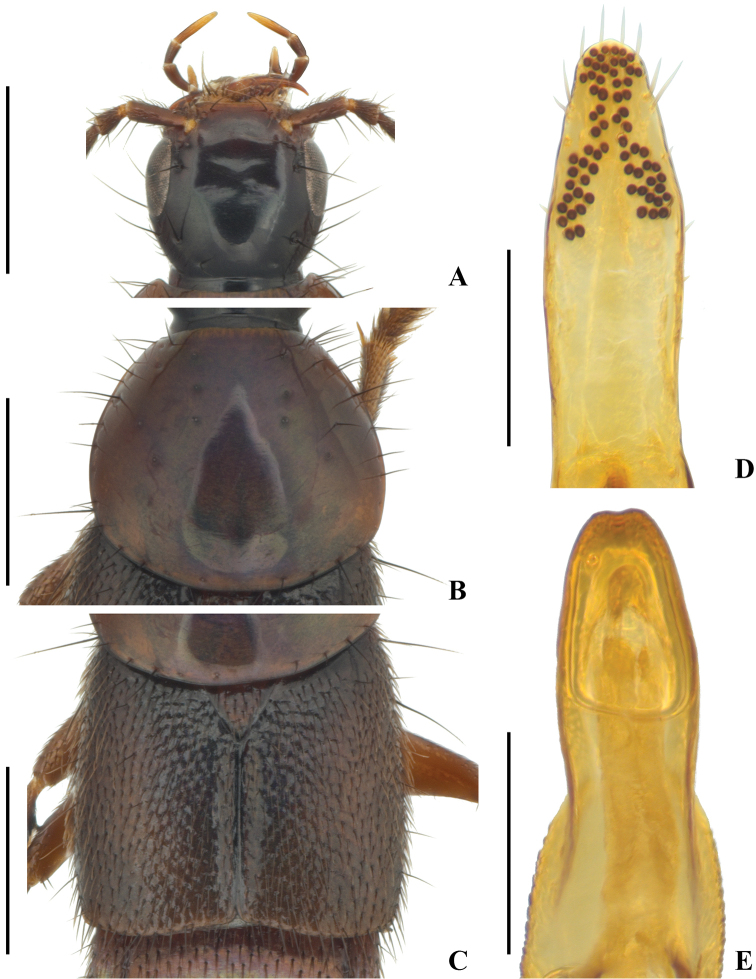
*Q. saetosum* sp. nov., morphology **A** head **B** pronotum **C** elytra **D** underside of paramere **E** apical portion of median lobe, parameral view. Scale bars: 1 mm (**A−C**); 0.20 mm (**D, E**).

Head (Fig. 1A) obtusely quadrangular, nearly as wide as long, HW/HL = 1.05; eye moderately large and slightly convex, in dorsal view tempora shorter than length of eye, gradually narrowed posteriad, HEL/HTL = 1.50; no additional setiferous punctures between anterior frontal setiferous punctures; posterior frontal setiferous puncture situated distinctly behind level of posteriomedial margin of eye, about midway between posteriomedial margin of eye and nuchal constriction of head; temporal setiferous puncture situated closer to posterior margin of eye than to nuchal constriction, with some small setiferous punctures behind and below it; one basal setiferous puncture situated closer to nuchal constriction than to posterior frontal setiferous puncture; head with very fine and dense microsculpture of transverse waves and meshes. Antenna moderately long, with segment I longer than segment II or III, segment III longer than segment II, segments IV–XI slightly longer than wide.

Pronotum (Fig. 1B) large and broad, wider than long, PW/PL = 1.10, distinctly narrowed anteriad, posteriolateral and posterior margins continuously and broadly rounded, lateral margins not explanate; three setiferous punctures in each dorsal and sublateral row, one additional setiferous puncture situated between each dorsal and sublateral row, distinctly behind level of last dorsal and sublateral row puncture, last sublateral row puncture situated behind level of large lateral setiferous puncture; one smaller additional setiferous puncture before each large lateral puncture; surface of pronotum with microsculpture similar to that of head, but even finer.

Scutellum (Fig. 1C) with dense setiferous punctures, surface with very fine and dense microsculpture of transverse waves.

Elytra (Fig. 1C) broad, slightly wider than long, EW/EL = 1.08, ESL/EL = 0.52, nearly parallel-sided laterally, each elytron with surface covered with dense setiferous punctures, transverse interspaces between punctures about as wide as diameter of punctures; surface between punctures with semigranulose microsculpture. Wings fully developed.

Abdominal tergite II finely punctate; setiferous punctures of other tergites finer and sparser than those of elytra, distinctly becoming sparser toward posterior margin of each tergite, and generally becoming so toward apex of abdomen; tergite VII with whitish apical seam of palisade setae.

**Figure 2.**
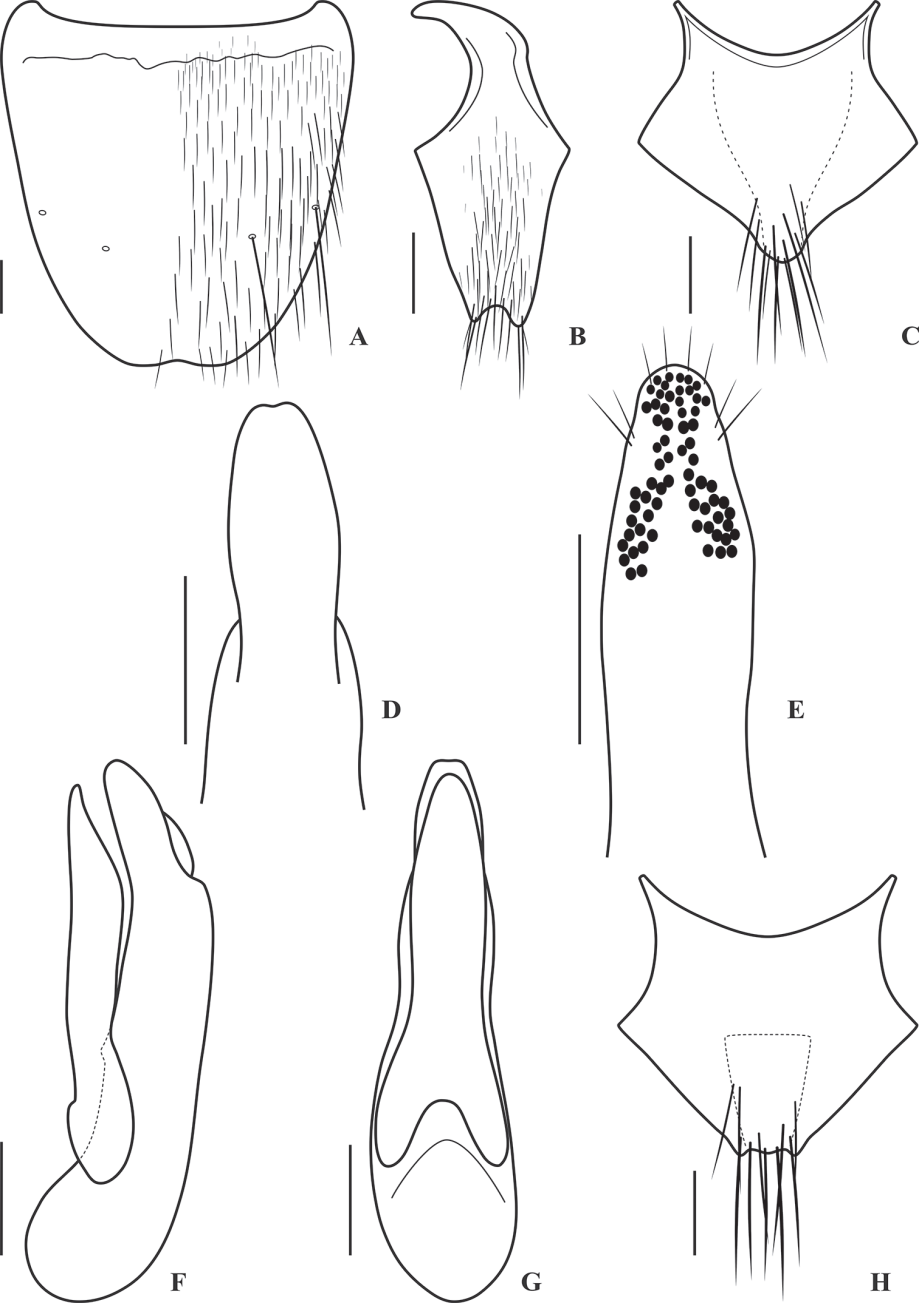
*Q. saetosum* sp. nov., morphology **A** male sternite VIII **B** male sternite IX **C** male tergite X **D** apical portion of median lobe, parameral view **E** underside of paramere **F** aedeagus, lateral view **G** aedeagus, parameral view **H** female tergite X. Scale bars: 0.20 mm.

Male with first four segments of foretarsus moderately dilated, sub-bilobed, each heavily covered with tenent setae ventrally, segment II slightly narrower than apex of tibia. Tergite VIII with basal ridge complete, slightly arched backward in middle, surface without long seta; sternite VIII (Fig. 2A) with basal ridge complete, slightly sinuate, with two long setae on each side, apical margin with very shallow and narrow medioapical emargination, a very small triangular area in front of the emargination impunctate; sternite IX (Fig. 2B) with basal portion long and curved, with a moderately deep arcuate medioapical emargination, with two long setae on each side of the emargination apically; tergite X (Fig. 2C) with basal side broadly and deeply concave, apical margin rounded, vaguely protruded. Aedeagus in lateral view (Fig. 2F) with apex of paramere not quite reaching that of median lobe, median lobe bent toward parameral side, without any process at apex; in parameral view (Fig. 2G) with paramere slightly narrower than median lobe, apical 1/4 gradually narrowed, forming rounded apex, median lobe wide at base, slightly narrowed in middle, distinctly constricted at about apical 1/5, apex subtruncate, with an inconspicuous medioapical emargination (Figs 1E, 2D); apical portion of paramere with four moderately long apical setae, and two similar subapical setae on each lateral side below apex, underside with numerous sensory peg setae arranged into one characteristic inversed Y-shaped figure at apex (Figs 1D, 2E).

Female first four segments of fore tarsus similar to those of male, but less dilated; sternite VIII with basal ridge inconspicuous, with 2 long setae on each side; tergite X (Fig. 2H) with basal side broadly and deeply concave, with subtriangular area in middle more strongly sclerotized and pigmented, apical margin incomplete, forming distinctive M-shaped indention.

#### Distribution.

**Figure 3.**
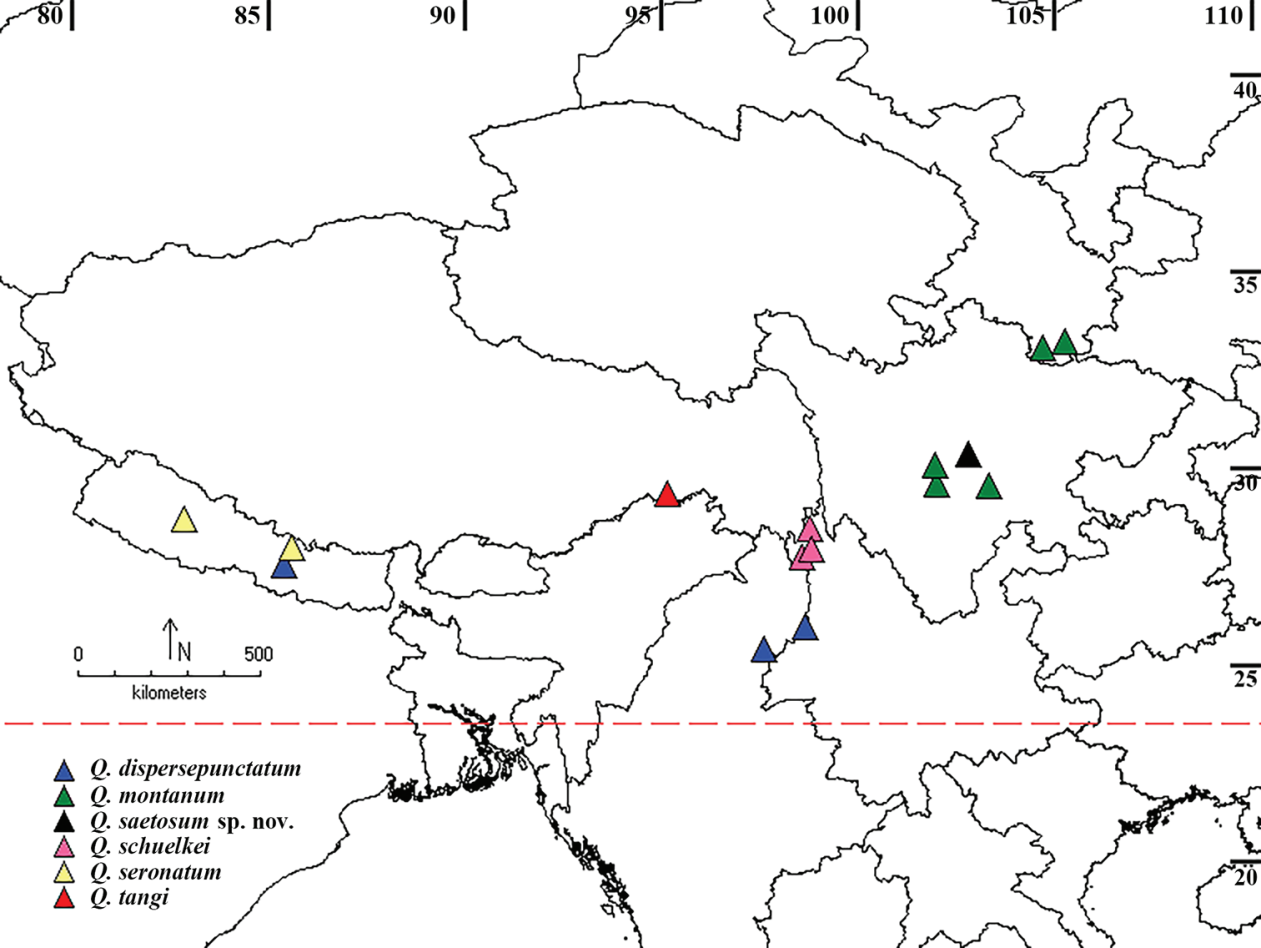
Known distribution of *Queskallion* species in China, Myanmar and Nepal.

*Queskallion saetosum* sp. nov. is at present known only from the type locality in central Sichuan Province (Fig. 3), China, at an altitude of 1600 m. The specimens were collected from stacks of withered grass on the roadside in August.

#### Etymology.

The specific name is from the Latin adjective *saetosus*, -*a*, -*um* (bristly), referring to the additional setae on the pronotum.

## Supplementary Material

XML Treatment for Queskallion

XML Treatment for Queskallion saetosum
